# High performance computing enabling exhaustive analysis of higher order single nucleotide polymorphism interaction in Genome Wide Association Studies

**DOI:** 10.1186/2047-2501-3-S1-S3

**Published:** 2015-02-24

**Authors:** Benjamin Goudey, Mani Abedini, John L Hopper, Michael Inouye, Enes Makalic, Daniel F Schmidt, John Wagner, Zeyu Zhou, Justin Zobel, Matthias Reumann

**Affiliations:** 1 IBM Research - Australia, 204 Lygon Street, Carlton, VIC, 3053, Australia; 2 Department of Computing and Information Systems, University of Melbourne, Parkville, VIC, 3010, Australia; 3 Melbourne School of Population and Global Health, University of Melbourne, Parkville, VIC, 3010, Australia; 4 Department of Pathology and Department Microbiology and Immunology, University of Melbourne, Parkville, VIC, 3010, Australia; 5 IBM Research Collaboratory for Life Sciences-Melbourne, 187 Grattan Street, Carlton, VIC 3010, Australia; 6 Department of Mathematics and Statistics, University of Melbourne, Parkville, VIC, 3010, Australia

## Abstract

Genome-wide association studies (GWAS) are a common approach for systematic discovery of single nucleotide polymorphisms (SNPs) which are associated with a given disease. Univariate analysis approaches commonly employed may miss important SNP associations that only appear through multivariate analysis in complex diseases. However, multivariate SNP analysis is currently limited by its inherent computational complexity. In this work, we present a computational framework that harnesses supercomputers. Based on our results, we estimate a three-way interaction analysis on 1.1 million SNP GWAS data requiring over 5.8 years on the full "Avoca" IBM Blue Gene/Q installation at the Victorian Life Sciences Computation Initiative. This is hundreds of times faster than estimates for other CPU based methods and four times faster than runtimes estimated for GPU methods, indicating how the improvement in the level of hardware applied to interaction analysis may alter the types of analysis that can be performed. Furthermore, the same analysis would take under 3 months on the currently largest IBM Blue Gene/Q supercomputer "Sequoia" at the Lawrence Livermore National Laboratory assuming linear scaling is maintained as our results suggest. Given that the implementation used in this study can be further optimised, this runtime means it is becoming feasible to carry out exhaustive analysis of higher order interaction studies on large modern GWAS.

## Background

Genome-wide association studies (GWAS) are a common approach for systematic discovery of genetic variants, typically single nucleotide polymorphisms (SNPs), which are associated with a given disease. Standard univariate analysis techniques, where each SNP is examined separately of all others, have detected novel regions of association in many diseases that were previously unknown [1]. Despite these findings, the total level of association between variants detected from GWAS and complex diseases is typically lower than the theoretical estimates of genetic heritability; the issue of "missing heritability" [2]. One common hypothesis is that the univariate approaches commonly employed may miss important associations that can only appear through multivariate SNP interaction analysis [3,4]. However, the computationally complexity of even the simplest interaction analysis, e.g. examining pairs of SNPs, grows exponentially compared to a univariate analysis.

The computational difficulties of exhaustive multivariate SNP analysis in GWAS has long been hampered by lack of computing resources [5]. Algorithmic improvements and improved processor speeds means that two-way interactions can currently be carried out in a few days [6-8]. Using graphics accelerators (GPUs) and parallel computing, the time to conduct this type of analysis can be reduced to hours for small to medium GWAS datasets [9-11]. However, to exhaustively search all SNP interactions containing three or more SNPs analysis increases the search space dramatically and exhaustive analysis of this task remains is currently infeasible [12]. For three-way interactions, the time using CPU based techniques has been estimated to take up to 1.5 million years [13] on a single processor computer. Even when using the fastest techniques using GPU cards, an examination of all three-way interactions would take years [10].

Supercomputing holds promise on delivering higher order interaction studies on exhaustive search in GWAS but have not yet been examined in depth. In this article, we explore how state-of-the-art methods for representing SNPs can leverage supercomputing systems to enable exhaustive multivariate analysis of GWAS data. Building on our previous work [14], we present a fast framework that allows evaluation of SNP interactions using any contingency table (CT) based statistical tests. We demonstrate the applicability of such a framework to high performance computing systems and demonstrate the potential that such systems may have to allow exhaustive analysis of higher-order interactions of three-way interaction studies on smaller GWAS sizes.

## Methods

### Notation

We denote each GWAS study as a collection of *m *SNPs and *n *samples. The class label, *Y*, for each sample is denoted as 0 for controls and 1 for cases. For diploid organisms, considered in most GWAS, each SNP can take one of three genotypes depending on whether the SNP variant occurs on zero, one or both copies of an individuals relevant chromosome. We denote these genotype values as 0,1 and, 2 respectively. For *k*-way interaction of SNPs, we have a set of possible genotype combinations *V *where the total number of possible genotype combinations, |*V*|, grows exponentially (3*^k^*) with the size of the interaction. For 2-way interactions (pairs), we have |*V*| = 3^2 ^= 9 possible genotype combinations while for 3-way SNP interactions (triples) we have |*V*| = 3^3 ^= 27 possible genotype combinations.

The discrete nature of the data in SNP interaction analysis, with two possible phenotype values and three possible genotype values per SNP, allows us to summarise the occurrence of a given SNP interaction as a contingency table. In Table 1 we describe such a table for an arbitrarily sized SNP interaction. Each cell indicates the occurrence of a specific genotype combination *v *∈ *V *in either cases or controls. We use the notation adopted in Agresti [15] to describe the table cells where *n_ij _*is used to denote the observed count in the cell (i, j). Marginal counts can be described using a standard plus convention, e.g., $n_{i+}=\underset{j}{\sum }n_{ij}$ is the occurrence of all genotypes for a given phenotype, *i*. The use of contingency table based analysis is common for GWAS studies as it allows for the application of a wide variety of statistical techniques [2].

**Table 1:** Example contingency table.

	Genotype Frequencies				
**Phenotype**	**1**	**2**	**..**.	**V**	**Row Counts**

Controls *Y *= 0	*n*_01_	*n*_02_	...	*n*_0*V*_	*n*_0+_

Cases *Y *= 1	*n*_11_	*n*_12_	...	*n*_1*V*_	*n*_1+_

Col. Counts	*n*_+1_	*n*_+2_	...	*n*_+*V*_	*n*

2 × *V*-contingency table summarising the occurrence of genotype combinations for an arbitrary SNP interaction in a case-control GWAS study.

### Framework for contingency table based interaction analysis

To conduct an exhaustive interaction analysis of an entire GWAS on a massively parallel supercomputer, we have implemented a fast contingency table-based framework using the high-level procedure described in Algorithm 1. It was implemented in the C/C++ programming language using the Message Passing Interface (MPI) for inter-node communication and OpenMP for intra-node parallelism.

After system initialization, during which the number of nodes and dimensions of the GWAS dataset are determined, the data decomposition and load balancing step establishes the workload for each process (MPI rank). The GWAS dataset is loaded onto each node using MPI broadcast so that each process can access it in memory. Each process then evaluates all allocated SNP interactions. For each interaction, we derive a contingency table describing the occurrence of each possible genotype combination in case and control samples for the given SNP interaction. A chosen statistic is then applied to the contingency table and a score, typically either a test statistic, a heuristic score or a *p*-value, is derived. This phase of the algorithm can be carried out in memory which allows the use of shared memory parallelism. After all possible interactions have been evaluated, the list of all recorded SNP interactions is sent back to the lowest rank node, and the combined set of significant SNP interactions are written to disk.

**input **: *X*: GWAS dataset with *m *SNPs × *n *individuals

            *k*: The order of interaction we wish to examine (2 = pairs, 3 = triplets etc.)

            *rank*: Rank of current compute nodes

            *nodes*: Total number of compute nodes

            *t*: significance threshold of given statistic

**output**: *significant_interactions *= List of SNP interactions that meet significance threshold

1 *significant_interactions *= {}

   // Determine the set of SNP interactions for this rank to evaluate

2 *allocated_interactions *= assignInteractions(*rank*, *m*, *k*)

   // Evaluate each possible tuple of SNP indices describing the interaction

3 **for ***indicies_tuple *in allocated_interactions**do**

4      *ct *= buildContingencyTable(*indicies_tuple*, *X*)

5      *score *= evaluateStatistic(*ct*)

6      **if ***score *≥ *t ***then**

7         *significant_interactions*.insert(*indicies_tuple*, *score*)

8      **end**

9 **end**

   // Nodes with a rank > 0 send recorded interactions to rank 0

10 **if ***rank > 0 ***then**

11      send(*significant_interactions*,0);

   // Rank 0 gets interactions from all nodes, merges them & saves them to disk

12 **else**

13      *significant_interactions *= receiveSignificantInteractions(*nodes*-1)

14      write(*significant_interactions*)

15 **end**

**Algorithm 1: **High level overview of framework for parallel GWAS interaction analysis. All nodes are allocated a set of SNP interactions to evaluate independent of all other processes. The lowest rank node is responsible for outputting the significant interactions to disk.

This type of algorithm has been commonly used by many of the fastest exhaustive SNP interaction analysis methods currently available [6,11,12,16]. The exhaustive phase modelled here is typically used to apply a fast screening statistic, with detected interactions then evaluated using a slower, more robust filter.

### Data representation and calculation of contingency tables

The analysis framework used in this work has two core steps to evaluate each interaction: building a contingency table of genotype combination occurrences and calculating a test statistic over the resulting table. Given the computational simplicity of many tests of association or interaction, often the computational bottleneck is the building of contingency tables for SNPs in a given interaction. The large number of interactions that exist for any exhaustive multivariate SNP analysis means that reducing the time to evaluate each SNP interaction is key to improving the overall system performance.

By taking advantage of the low number of possible genotype values, some implementations [6,12,16] have been able to use a binarisation of individual SNPs to form a representation that simultaneously reduces the space taken by each genotype to two bits, while enabling parallel evaluation of multiple samples in a single operation.

**Figure 1:**
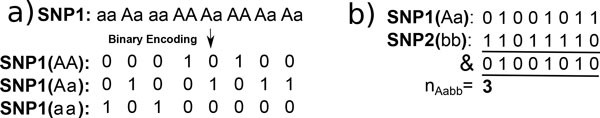
**Binary genotype representation**. Example showing a) the conversion of a given SNP into the binary representation, b) computing the occurrence of a single genotype combination for a pairs of SNPs by taking their logical AND and counting the number of set bits in the resulting binary vector.

Each SNP can be viewed as a vector containing up to three values, four if "missing" genotypes are stored. Rather than representing this SNP as a single vector of three values, we can instead represent the SNP as three separate binary vectors; one for each of the three possible genotype values. Each binary vector has a '1' to indicate samples containing the relevant genotype, and a '0' for the remaining samples. An example showing the transformation of genotype data for a single SNP to this binary representation is shown in Figure 1a.

Storing each genotype as a separate binary vector not only reduces the space required to store each genotype but also allows for calculation of contingency tables using fast bitwise operations. Consider the case of evaluating the co-occurrence of a given genotype combination for a SNP pair, illustrated in Figure 1b. Taking the logical AND of the two relevant binary vectors, indicates which samples the genotypes co-occur in. Counting the number of set bits in the resulting binary vector, provides the total number of times the given genotype combination occurs. These two operations, logical AND and counting the set bits, can both be computed over a 64-bit word using only two hardware operations for most modern CPUs. Repeating this computation for all genotype combinations in case and control samples allows us to quickly compute the contingency table for each SNP pair. Such an operation is trivially extended to genotype combinations for any *k*-way interaction of SNPs.

### Pearson's Chi-squared test

There exists numerous tests of association that can be implemented in our interaction analysis framework. In this work, focussing on the computational rather than statistical, aspects of GWAS we have implemented Pearson's chi-squared test. The chi-squared test is perhaps the most commonly used test to determine whether two sets of paired observations are statistically independent [17]. In the context of GWAS studies, the test is used to assess departure from the null hypothesis that the distribution of genotype combinations is the same for case and controls. While other statistics for testing independence exist, Pearson's chi-squared test has been previously used in SNP interaction analysis techniques [7,18-20] because it is simple to compute and is easy to interpret [15]. The closed form solution of Pearson's chi-squared test means its computation is far faster than methods that derive solutions through iterative methods, such as logistic regression [8], or methods that require repeated simulations, such as many Bayesian approaches [21].

Given the contingency table for a specific SNP interaction, the test statistic for the chi-squared test can be written as

(1) $$\chi^{2}=\overset{2}{\underset{i=1}{\sum }}\overset{\left|V\right|}{\underset{j=1}{\sum }}\frac{{\left(n_{i,j}-E_{i,j}\right)}^{2}}{E_{i,j}}$$

where $E_{i,j}=\frac{n_{i+}\cdot n_{+j}}{n}$, *n*_*i*+ _is the total number of samples in phenotype class *i*, *n*_+*j *_is the total samples carrying a specific genotype *j *∈ *V *, and *n *is the total number of samples.

A *p*-value can be derived by the comparing the resulting test score to the chi-squared distribution with degrees of freedom, *df *= |*V*| − 1.

### Data decomposition and load balancing

As with many other exhaustive SNP interaction analysis methods, each SNP interaction in our framework is evaluated with no dependency on any other SNP interaction. This independence of evaluation means that the data decomposition is "pleasantly parallel" and is therefore particularly suitable for distributed computing implementation.

To divide the total set of interactions amongst an arbitrary set of computation processes, we first determine the total number of interactions that need to be evaluated. For a given GWAS data set with *m *SNPs, the total number of $\left(\begin{array}{c} m\\ k \end{array}\right)=\frac{m!}{k!\left(m-k\right)!}$ interaction terms. Dividing this total by the number of parallel processes, *N_P _*yields the number of interactions to be tested by each process,

(2) $$Interactions_{process}=\left(\begin{array}{c} m\\ k \end{array}\right)/N_{P}$$

where *N_P _*is the total number of parallel processes used during the computation *i.e*. $N_{P}=N_{core}\times N_{threads}$. Each test can be considered to have an index in a one-dimensional array of tests. Thus, each process knowing its MPI rank, the indices for the lower and upper bounds for each process within the overall test array can be defined within each process independently.

To determine which SNP interactions correspond to the respective test, we can make use of combinatorial number systems. This allows for conversion of a given SNP *k*-way interaction to a linear index between 0... $\left(\begin{array}{c} m\\ k \end{array}\right)\text{ - }1$ by applying the following formula

(3) $$k\text{-}way_{index}=\left(\begin{array}{c} x_{k}\\ k \end{array}\right)+\cdot \cdot \cdot +\left(\begin{array}{c} x_{2}\\ 2 \end{array}\right)+\left(\begin{array}{c} x_{1}\\ 1 \end{array}\right)$$

where (*x*_k_, ..., *x*_2_, *x*_1_) represents an *k*-way interaction where *x *represents the indices of the SNPs in the data set whose interaction is being tested. Note that the condition *x_k _*> ... >*x*_2 _>*x*_1 _must be met otherwise there will be duplication of testing for interaction terms. For example in a 3-way interaction study, a given triple of SNPs (*x*_3_, *x*_2_, *x*_1_) would become the linear index

(4) $$3\text{-}way_{index}=\left(\begin{array}{c} x_{3}\\ 3 \end{array}\right)+\left(\begin{array}{c} x_{2}\\ 2 \end{array}\right)+\left(\begin{array}{c} x_{1}\\ 1 \end{array}\right)$$

such that *x*_3 _>*x*_2 _>*x*_1_.

**Figure 2:**
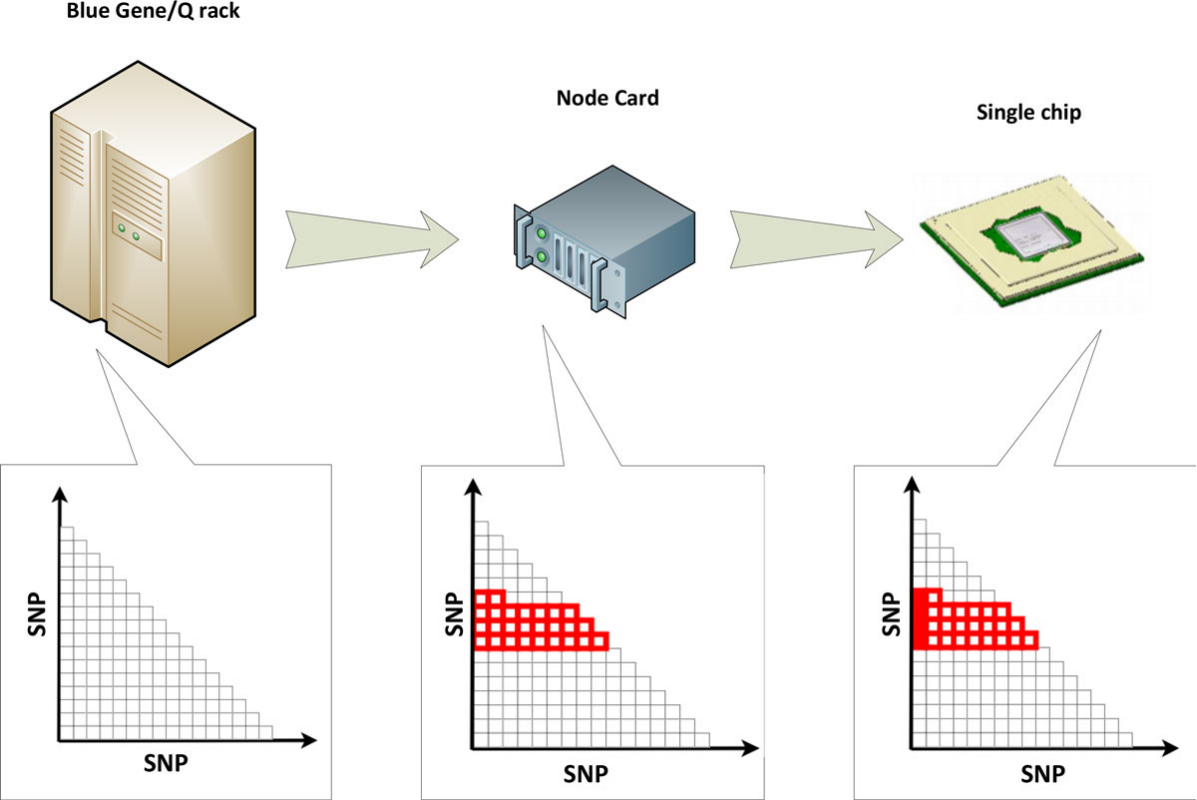
**Illustration of data decomposition and load balancing**. Decomposition strategy. For any given SNP interaction study, the entire calculation is divided into equal-sized partitions. For each partition one MPI task is executed on an assigned CPU card. The further decomposition into small sub-tasks are handle by OpenMP dynamic scheduler.

This simple calculation using overall workload, *i.e*. the total number of interaction tests to be computed, and the process identifier (MPI rank) of a given node then gives us the set of interaction terms that need to be processed by the specific node. By leveraging shared memory parallelism using the OpenMP application programmers interface, the set of interactions to be examined by each node are further parallelized across different hardware threads. The multiple levels of decomposition and parallelism are illustrated in Figure 2.

### Computational resources

The computing resources available for the presented study comprise an IBM Blue Gene/Q (BG/Q) supercomputer named "Avoca" hosted by the Victorian Life Science Computation Initiative (VLSCI). Avoca is a four-rack BG/Q installation with 4,096 compute nodes. Each node is a 64-bit system-on-chip POWER A2 processor with 16 cores dedicated to computation and one supplemental core to handle operating system tasks. Each core supports four-way hardware threads. Thus, Avoca comprises 65,536 compute cores with support for 262,144 hardware threads. BG/Q can be set up during initialization such that each physical node can run a single process (MPI rank) with 16 × 4 threads (four for each core on each node). The ratio of processes per node versus threads per process can be changed in orders of two to the other extreme where each hardware thread can run a single process with a single thread each. Depending on the given set up, memory per process changes. The processors are clocked at 1.6 GHz and have 16 GB per compute node of directly accessible memory. The four-rack BG/Q therefore has a total of 64 TB of distributed memory. The communication network that links all compute nodes is a five-dimensional torus that enables very high bandwidth and fast communication.

While largely different in size and technology, all supercomputing systems have the characteristic of being massively parallel, distributed memory supercomputers. The parallel, distributed memory characteristic also applies to local or cloud based clusters. Hence, our methodology is equally applicable to any parallel computer, both with distributed and shared memory.

### Simulated datasets

In order to examine the time efficiency for the data representations discussed in this work, we have simulated human genotype data using the HAPGEN2 software [22]. Data is simulated by making use of known haplotype blocks, *i.e*. sequences of (DNA) on the same chromosome that are typically inherited together. Given a reference panel of known haplotype blocks, such as that from HapMap [23] or 1000 Genomes Project [24], the HAPGEN2 method resamples sequences of haplotype blocks from across the reference panel to generate a new "imperfect mosaic" of DNA sequence, mimicking the effect of recombination [22]. By resampling from real human data, the technique maintains the linkage disequilibrium structure, i.e. correlations between local SNPs, seen across the genome. Using the HAPGEN2 software combined with the 1000 Genomes reference panel, we generated datasets of varying sizes ranging between 100 to 1.1 million SNPs, each with 2000 samples.

### Measures of runtime performance

To evaluate the parallel performance, we have carried out strong scaling simulations and consider the speedup and efficiency of each run as the main measures by which we evaluate our implemented framework. We define the speedup *S *of the program as

(5) $$S=\frac{t_{p=1}}{t_{p=N}}$$

with *t*_*p *= 1 _being the measured time in serial execution versus the measured time on *N *threads *t*_*p *= *N*_. Based on the speedup, we can define the efficiency *E *as

(6) $$E=\frac{S}{N}=\frac{t_{p=1}}{N_{P}\times t_{p=N}}$$

A program is called "strongly scalable" if by increasing the number of parallel threads *p*, we can get a constant efficiency *E *while problem size remains fixed. Thus, strong scalability is indicative of the efficient use of parallel computing resource.

We can break this down to evaluate the scaling factor that appears between consecutive runs with different numbers of hardware threads. If our program is strongly scalable, the total run time should be decrease by a factor of two if the number of processors is increased by factor two.

### Scaling of previously reported timings

A comparison of runtime for the framework presented in this study with that of previously reported SNP interaction analysis techniques is difficult given that reported timings were run over different hardware. Lack of availability of source code for many methods means that we cannot port these algorithms directly to Blue Gene. Instead, we have estimated the running times from the times reported in literature when scaled to the same hardware and dataset size. For CPU-based methods, we have scaled times to those of the Avova BG/Q system with 262,144 threads running at 1.6 Ghz. For GPU-based methods, we have scaled times to those of the Nvidia GTX470 graphics card with 448 hardware threads running at 1.22 GHz. Both estimated timings were also scaled to estimate runtime over a datasets containing 2000 samples and 1.1 million SNPs, as used in 3. For all methods we assume prefect linear scaling with time and number of interactions. Using these criteria, the column for "Scaled" runtime in Table 2 is obtained using the following formulas.

**Table 2:** Hardware, dataset size and previously reported runtimes for a variety of CPU- and GPU-based pairwise SNP analysis methods.

Method	Hardware	Cores	Clock (Ghz)	SNPs (1000 s)	Samples (1000 s)	Time (min)		Estimated 3-way runtime
						Unscaled	Scaled	
CPU-based								

Present method	PowerPC A2	262,144	1.60	1,100	2	8	8	5.8 years

Wang	Intel X3430	172	2.40	500	2	538	3	>1000 years

Ma	Intel X5355	528	2.66	50	2	1158	1877	>1000 years

GPU-based								

Goudey	Nvidia GTX470	448	1.22	450	5	13	31	21.7 years

GBOOST	Nvidia GTX285	240	1.48	351	5	80	220	219 years

Kam-Thong	Nvidia GTX295	960	1.24	4	10	2	19664	>1000 years

Hardware, dataset size and previously reported runtimes for a variety of CPU- and GPU-based pairwise SNP analysis methods. For each method, we show the make and model of processor or GPU card, the processor speed and the number of hardware cores. Runtime is additionally shown as originally reported as well as scaled to a dataset with 1.1 million SNPs, 2000 samples and using either the full Avoca system (CPU methods) or using the Nvidia GTX295 (GPU methods). In the final column we show the estimated time to processes all 3-way interactions for a 1.1 million SNP, 2000 sample dataset using the originally reported hardware and assuming perfectly linear scaling.

Formula for CPU scaling estimate (1.1 million SNPs, 2000 samples, 262,144 threads running at 1.6 Ghz)

(7) $$t_{CPU}=t_{orig}\times {\left(\frac{1.1\times 10^{6}}{n_{SNP}}\right)}^{2}\times \frac{2000}{n_{sample}}\times \frac{n_{cores}}{262144}\times \frac{f_{clock}}{1.6}$$

Formula for GPU scaling estimate (1.1 million SNPs, 2000 samples, 448 threads running at 1.22 Ghz)

(8) $$t_{GPU}=t_{orig}\times {\left(\frac{1.1\times 10^{6}}{n_{SNP}}\right)}^{2}\times \frac{2000}{n_{sample}}\times \frac{n_{cores}}{448}\times \frac{f_{clock}}{1.22}$$

where *t_orig_*, *N_sample_*, *N_SNP_*, *N_cores _*and *f_clock _*is the runtime, number of samples, SNPs, threads and clock frequency reported in the original study.

The estimates obtained by these scaling formula are rough given there are many differences in hardware that have not been taken into account. Nevertheless, these estimated times should provide an indication of difference in runtime performance between algorithms.

## Results and discussion

### Threads vs time

To explore the strong scaling of the runtime of our framework with respect to the number of parallel hardware threads used, we have evaluated the runtime of our framework as the number of hardware threads is exponentially increased from 128 (2^7^), the lowest allocation on the Avoca system that can hold the dataset in memory, to 262,144 threads (2^18^), the entire Avoca system. For each run, we fixed the dataset size at 1.1 million SNP and 2000 samples, the largest set of simulated data we have generated. Each run evaluates over 605 billion SNP pairs. We record the total time required to exhaustively evaluate all SNP pairs as the number of threads is altered, including overhead such as communication between threads and data loading time.

**Figure 3:**
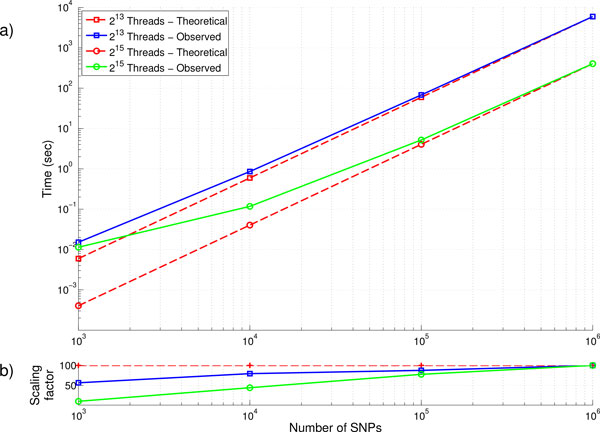
**Run time, scaling and efficiency analysis for strong scaling simulations**. Total run times, scaling and efficiency (a., b. and c. respectively) as the number of hardware threads is increased for a 1.1 million SNP, 2000 sample dataset.

Using the full Avoca system, we are able to evaluate 605 billion SNP pairs in less than 10 minutes. The time to compute the test statistic on the whole GWAS dataset as the number of hardware cores increases is shown in Figure 3a. It shows observed runtime as well as the theoretically time required in the case of perfect scaling. The scaling factor, the speed-up in computation time as the number of threads is increased, between cores is shown in Figure 3b. We show efficiency, as calculated in Equation 6, in Figure 3c starting with a baseline runtime measured at 128 (2^7^) hardware threads.

Scaling is almost linear for fewer than 2^12 ^cores and more than 2^14 ^with a drop in scaling efficiency at 2^13 ^cores. The drop in efficiency may be caused due inefficiencies in our code with respect to translation onto the physical hardware system including IO cards and torus topology. Aside from the discrepancy in timing results seen at 2^13 ^cores, we find that the runtime is almost halved as the number of hardware threads is doubled. The cumulation of discrepancies in the scaling factor reveals that the overall efficiency of the system drops to almost 50% as the framework is scaled up to the maximum number of hardware threads. However, the loss in efficiency is due to the inefficiency going from 2^13 ^to 2^14 ^hardware threads and is carried forward. Since the efficiency and scaling factor continues to be constant from 2^14 ^hardware threads and higher, one can argue that parallel efficiency will be close to 100% with code/hardware optimization to remove the discrepancy at 2^13 ^hardware threads. Resolving these reductions in scaling efficiency indicates that the runtime required to evaluate all SNP pairs in a 1.1 million SNP dataset should drop below 5 minutes if using Avoca's full 2^18 ^threads.

### SNP vs time

Similarly, we can evaluate the scaling of runtime as the problem size *i.e*. the number of SNPs is varied, while the number of hardware threads is kept constant. Given that the 1.1 million SNP dataset used in Figure 3 is larger than the majority of current GWAS studies, the time required for smaller datasets may be significantly smaller and require less hardware. We have therefore generated subsets of our simulated data at increasing powers of 10 (10^3 ^- 10^6^) and evaluated these using two different amounts of threads; 8192 (2^13^) and 31768 (2^15^), representing 3.1% and 12.5% of Avoca's total system size respectively. For each of these runs, we have again recorded the total runtime and evaluated the scaling factor between each decrease in datasets size.

**Figure 4:**
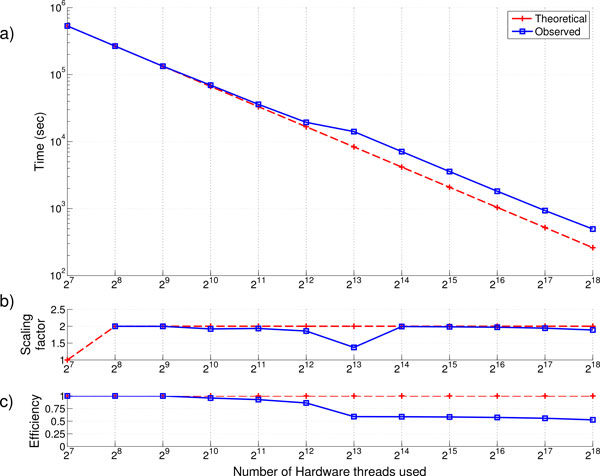
**Run times and scaling for varying size GWAS datasets on 64 and 1024 computing nodes**. Runtime and scaling (a. and b. respectively) as the number of SNPs increases, using either 1024 or 64 nodes respectively. Subsets of the simulated data at increasing powers of 10 (10^3 ^- 10^6^) are used. The scaling factor in subplot b. indicate the decrease in runtime as the number of pairs is reduced by a factor of 100.

When the size of the dataset being evaluated has greater than 100,000 SNPs (5 billion SNP pairs), Figure 4a shows that the scaling of runtime is almost linear with the number of SNP pairs. This is confirmed by examination of the scaling factor in Figure 4b. For these larger datasets, as the number of SNP is reduced by 10 and the corresponding number of SNP pairs and the overall runtime is reduced by a factor of 100.

Given that the number of pairs increases quadratically with the datasets size, smaller datasets require far less computation to conduct the interaction analysis. The time require for analysis rapidly drops below a few seconds for datasets with fewer than 10,000 SNPs (50 million SNP pairs). In these datasets, the runtime required to process evaluate the SNP pairs becomes so low that communication overhead and memory allocation begin to take a large enough percentage of time for each node and impact scaling efficiencies. This can be observed to a greater degree as a larger number of hardware threads are used, decreasing the amount of work that is required by each individual process. Given that the runtime for these is less than a few seconds, the practical impact of this drop in efficiency is low.

### Comparisons with previous interaction techniques

The inherent parallelism in exhaustive mutivariate GWAS analysis allows this type of analysis to be conducted over a variety of hardware. In this work, we have developed a flexible framework for interaction analysis in GWAS studies and scaled our analysis to the full Avoca Blue Gene/Q system. However, other solutions using parallel CPU and GPU based methods have been previously conducted. To address the computational challenge of exhaustive GWAS analysis, Ma *et al*. (2008) [13] used the supercomputing facilities at the Minnesota Supercomputer Institute, an SGI Altix XE 1300 Linux cluster system with 2.66 GHz quad-core Intel X3430 processors with 2 GB of memory. The analysis was carried out on 132 compute nodes, *i.e*. 528 cores. Wang *et al*. (2011) [7] proposed a cloud-based solution, using a similar framework to that used in this study, over a 43 compute node cluster with 2.4 Ghz quad-core Intel X3430 processors with 8 GB of memory for investigating scalability of their method. GPU based methods have become popular for conducting this type of analysis given their increasing availability in desktop computers and every-increasing importance for scientific computing [25]. Methods by Goudey *et al*. (2013) [11] and Wan et al. (2010) [6] have used two-stage filtering methods, employing a fast analytical filter followed by a more computationally expensive filter. Kam-Thong *et al*. (2011) [9] conducted a similar analysis using a fast approximation to linear regression. All three GPU methods described above use Nvidia GPU cards to conduct their analyses.

We have summarised the hardware, data-size and runtime of all of these methods alongside the times reported reported in this study in Table 2. The hardware utilised by these different studies is clearly different with Ma *et al*. (2008) using only 0.1% of the threads that are avaliable in the full Avoca system. This difference in hardware is also clear for the GPU methods, which all used Nvidia graphics cards that vary greatly in the number and processing speed of these hardware threads.

It is unclear how the runtime of the various algorithms compare if the hardware and datasets sizes were made comparable. Therefore, we additionally report a scaled runtime, where an estimate of runtime is made if the dataset size is scaled to 1.1 million SNPs, 2000 samples and the number of processors and clock speed is scaled to be consistent with the full Avoca system for CPU methods or the Nvidia GTX295 for GPU methods. The formulas for this scaling is shown in the Methods section. In the final column, we show the estimated time to processes all 3-way interactions for a 1.1 million SNP, 2000 sample dataset based on the unscaled runtime (e.g. using original hardware) and assuming perfectly linear scaling.

The scaled timings indicate there may be some room for optimisation of the framework developed in this work, though it is competitive with state-of-the-art methods. The analysis techniques proposed by Wang *et al*.(2011) show a marked improvement over the times we report, and may indicate that there are further optimisations that can be applied to the engineering behind the framework we have presented here. Given the roughness of the runtime estimates conducted here, it is unclear how much of this improvement relates to factors not taken into consideration as part of the scaling conducted here.

The timings reported by the GPU methods examined here indicate that the use of GPU based systems is competitive with the runtime reported over the supercomputer. While GPUs are more commonly available and cheaper, from a research perspective there are some issues in developing novel algorithms for these cards. Improvement in speed depend on how much the communication between main memory and GPU memory can be reduced. The level of speed in communicating between these two memory caches is slow compared to the processor speed of the actual GPU cores. There is also only a small amount of memory available to each separate GPU core. These constraints have two large ramifications on the programming of GPU accelerated software. The first is that efficient programming of GPU-accelerated software requires a deep level of understanding about the underlying hardware architecture. Without understanding the different levels of memory and the ways to make efficient use of the memory registers and caches on the card, the level of improvement in speed is often small [26]. Secondly, the overall algorithm that is being optimised on the GPU must be full designed before any programming can begin. Alterations of the algorithm mid-way through implementation can have a large impact on the way that memory is allocated across the different registers in the card and hence can cause dramatic reduction in the level of speed-up gained [26]. The inflexibility of programming for GPU cards can limit the suitability of this type of hardware to research techniques that may be require ongoing improvements and optimisations. The supercomputer environments considered here are typically a development environment more similar to standard desktop programming, allowing for greater flexibility and a lower entry barrier to developing on them.

If we consider exhaustive search of three-way interactions, the results in Table 2 indicate that all methods on hardware examined here are too slow. An exhaustive three-way interaction analysis on 1.1 million SNP dataset would require over 5.8 years on the full Avoca machine. This is hundreds of times faster than estimates for other CPU based methods and four times faster than runtimes estimated for GPU methods, indicating how the improvement in the level of hardware applied to interaction analysis may alter the types of analysis that can be performed. On the currently largest IBM Blue Gene/Q installation "Sequoia" at Lawrence Livermore National Laboratory (24 times larger than Avoca with roughly 6.29 million computational threads), the same analysis could take only under three months assuming linear scaling. Given that the implementation used in this study can be further optimised, this runtime means it is becoming feasible to carry out exhaustive analysis of higher order interaction studies on large modern GWAS.

## Conclusion

This study illustrates that higher order interaction studies on GWAS data are becoming feasible on supercomputing systems. We have shown near-linear scalability of runtime with the number of threads on a parallel, distributed memory supercomputer allows for a reduction in analysis runtime that has not been achieved previously. While the approach taken here is yet to be applied over real data, the presented framework gives technical capability to enable previously unachievable research opportunities in GWAS analysis.

## List of abbreviations

GWAS - Genome Wide Association Studies

SNP - Single Nucleotide Polymorphism

GPU - Graphics Processing Unit

CPU - Central Processing Unit

CT - Contingency table

MPI - Message Passing Interface

*df *- Degrees of freedom

BG/Q - IBM Blue Gene/Q

VLSCI - Victorian Life Science Computation Initiative

GHz - Gigahertz

GB - Gigabyte

TB - Terabyte

DNA - Deoxyribonucleic acid

## Competing interests

The authors declare that they have no competing interests.

## Authors' contributions

Benjamin Goudey built the computational framework, implemented the test statistics and drafted the manuscript. Mani Abedini implemented the OpenMP functionality, conducted performance simulations and drafted the manuscript. Michael Inouye supervised the creation of the simulated GWAS data set. John L Hopper, Enes Makalic and Daniel F Schmidt, John Wagner and Justin Zobel contributed to project initiation funded by National Health and Medical Research Institute, Australia. All provided useful feedback in the manuscript preparation. Zeyu Zhou created the simulated data set and contributed to the performance simulations. Matthias Reumann supervised the overall project implementation with particular input to framework and implementation on the supercomputer as well as drafted the manuscript. All authors read and approved the final manuscript.

## Declaration

This research was partially funded by NHMRC grant 1033452 and was supported by a Victorian Life Sciences Computation Initiative (VLSCI) grant number 0126 on its Peak Computing Facility at the University of Melbourne, an initiative of the Victorian Government, Australia.

This article has been published as part of *Health Information Science and Systems *Volume 3 Supplement 1, 2015: Proceedings of the Health Informatics Society of Australia Big Data Conference (HISA 2013). The full contents of the supplement are available online at http://www.hissjournal.com/supplements/3/S1/
